# TSPO imaging using the novel PET ligand [^18^F]GE-180: quantification approaches in patients with multiple sclerosis

**DOI:** 10.1186/s13550-017-0340-x

**Published:** 2017-10-26

**Authors:** Lena Vomacka, Nathalie Lisa Albert, Simon Lindner, Marcus Unterrainer, Christoph Mahler, Matthias Brendel, Larissa Ermoschkin, Astrid Gosewisch, Anika Brunegraf, Christopher Buckley, Tania Kümpfel, Rainer Rupprecht, Sibylle Ziegler, Martin Kerschensteiner, Peter Bartenstein, Guido Böning

**Affiliations:** 1 Department of Nuclear Medicine, University Hospital, LMU Munich, Marchioninistr. 15, 81377 Munich, Germany; 2 Institute of Clinical Neuroimmunology, University Hospital, LMU Munich, Munich, Germany; 3 GE Healthcare, Grove Centre, Amersham, UK; 4 Department of Psychiatry and Psychotherapy, University of Regensburg, Regensburg, Germany; 5 Munich Cluster for Systems Neurology (SyNergy), Munich, Germany

**Keywords:** PET, [^18^F]GE-180, Multiple sclerosis, TSPO, Quantification

## Abstract

**Background:**

PET ligands targeting the translocator protein (TSPO) represent promising tools to visualise neuroinflammation. Here, we analysed parameters obtained in dynamic and static PET images using the novel TSPO ligand [^18^F]GE-180 in patients with relapsing remitting multiple sclerosis (RRMS) and an approach for semi-quantitative assessment of this disease in clinical routine.

Seventeen dynamic [^18^F]GE-180 PET scans of RRMS patients were evaluated (90 min). A pseudo-reference region (PRR) was defined after identification of the least disease-affected brain area by voxel-based comparison with six healthy controls (HC) and upon exclusion of voxels suspected of being affected in static 60–90 min p.i. images. Standardised uptake value ratios (SUVR) obtained from static images normalised to PRR were correlated to the distribution volume ratios (DVR) derived from dynamic data with Logan reference tissue model.

**Results:**

Group comparison with HC revealed white matter and thalamus as most affected regions. Fewest differences were found in grey matter, and normalisation to frontal cortex (FC) yielded the greatest reduction in variability of healthy grey and white matter. Hence, FC corrected for affected voxels was chosen as PRR, leading to time-activity curves of FC which were congruent to HC data (SUV_60–90_ 0.37, *U* test *P* = 0.42). SUVR showed a very strong correlation with DVR (Pearson ρ > 0.9). Focal MS lesions exhibited a high SUVR (range, 1.3–3.2).

**Conclusions:**

This comparison with parameters from dynamic data suggests that SUVR normalised to corrected frontal cortex as PRR is suitable for the quantification of [^18^F]GE-180 uptake in lesions and different brain regions of RRMS patients. This efficient diagnostic protocol based on static [^18^F]GE-180 PET scans acquired 60–90 min p.i. allows the semi-quantitative assessment of neuroinflammation in RRMS patients in clinical routine.

## Background

The classic diagnosis of multiple sclerosis (MS) is based on clinical and paraclinical documentation of the dissemination of CNS lesions in time and space. Such lesions and their evolution over time are commonly detected by magnetic resonance imaging (MRI). This forms not only the basis of the diagnosis but is also used to monitor disease activity and inform the decision on appropriate therapeutic strategies. While in MRI, the disruption of the blood-brain barrier (BBB) is used as proxy of disease activity, positron emission tomography (PET) imaging of activated microglia or macrophages with the 18-kDa translocator protein (TSPO) visualises one of the hallmarks of neuroinflammation and thus might provide a more direct approach to assess disease activity in MS. TSPO is primarily expressed in activated microglia, astrocytes, endothelial cells, and infiltrating macrophages [1] and is therefore associated with nervous system inflammation [2]. The prototypic TSPO radioligand [^11^C](R)-PK11195 has been frequently investigated in various PET imaging studies [3]. However, quantification with [^11^C](R)-PK11195 has been shown to be challenging due to a low free fraction in plasma, a significant binding to plasma proteins, and a low extraction fraction in brain with a limited signal-to-background ratio [4, 5]. This led to the development of second-generation TSPO radioligands with lower non-specific binding and higher affinity and specificity.

Preclinical data of the third-generation TSPO radioligand [^18^F]GE-180 have demonstrated a higher specific signal in affected brain regions and a lower non-specific binding in healthy tissue than [^11^C](R)-PK11195 in models of stroke [6] and neuroinflammation [7, 8]. Our own preclinical experience with this tracer indicated a very good applicability for monitoring neuroinflammatory disease as well [9]. First-in-human studies with healthy controls (HC) found a low first-pass extraction resulting in low uptake of [^18^F]GE-180 in healthy tissue [10, 11]. Various compartmental models with and without an extravascular component that takes into account tracer binding to endothelial cells were investigated and the authors suggested a two-tissue compartment model without an extravascular component as the preferred method for [^18^F]GE-180 quantification in healthy controls and 90 min as the optimal scan length for reliable estimation of volumes of distribution (*V*_T_) [10, 11]. Distribution volumes from Logan plot and semi-quantitative SUVs (60–90 min p.i.) correlated well with *V*_T_ from 2TC model [10, 11]. Although the so far available pre-clinical data are promising, the performance of [^18^F]GE-180 as a tracer for neuroinflammatory diseases in human patients still needs to be verified.

This is the first study investigating relapsing-remitting MS (RRMS) patients with [^18^F]GE-180 PET with the aim of quantifying the uptake in various anatomical brain regions and in focal lesions. In particular, we focused (1) on the identification of a pseudo-reference region (PRR), which is challenging in diseases with widespread inflammation within the brain [12], and (2) on the comparison of parameters obtained from dynamic and static data, the latter one avoiding long scan times and demanding data processing steps, with the goal of providing a quantification procedure which is suitable for routine clinical use.

## Methods

### Radiochemistry

As described previously [13], [^18^F]GE-180 production was performed on a FASTLab synthesiser with single-use disposable cassettes manufactured by GE Healthcare (The Grove Centre Amersham, UK). Radiochemical purity exceeded 95% and a high-specific activity was reached, ranging between 2423 and 3293 GBq/μmol.

### DNA extraction and polymorphism genotyping

Due to the reported dependency of binding properties of the second-generation TSPO ligands on a genetic polymorphism of the TSPO gene, all individuals were genotyped and classified as low-, medium-, or high-affinity binder (LAB, MAB, and HAB) [14–17]. Genotyping for TSPO polymorphism was performed at the Department of Psychiatry of the University Hospital Regensburg on 4 mL whole blood samples. Genomic DNA was extracted with QIAmp DNA blood maxi kit (Qiagen, Hilden, Germany) following the manufacturer’s protocol. DNA quality assessment was performed with optical absorbance and gel electrophoresis. Exon 4 of the TSPO gene containing the polymorphism rs6971 (Ala or Thr at position 147) as well as exon/intron junctions were PCR amplified and sequenced using Sanger method with the primers ex4-F-AGTTGGGCAGTGGGACAG and ex4-R-GCAGATCCTGCAGAGACGA. Sequencing data were analysed using SnapGene software (GSL Biotech; available at snapgene.com). The identified rs6971 genotypes (C/C, C/T, or T/T) code for the amino acids Ala/Ala, Ala/Thr, or Thr/Thr at position 147 of the TSPO protein and were considered to generate a high-, medium-, or low-affinity binding phenotype, respectively [17].

### Patient data and human subjects

Seventeen dynamic PET scans were performed in 14 RRMS patients (7 female and 7 male; mean age 39 ± 9 years; 5 MAB and 9 HAB). At the time of the PET scan, 4 patients were without treatment, 5 patients were receiving rituximab, 3 patients were receiving glatirameracetate, 2 were receiving natalizumab, and 1 patient each was treated with alemtuzumab, interferon-beta, and teriflunomide, respectively. The study with patients was approved by the local ethics committee (IRB no. 601–16) and the German radiation protection committee. All patients gave written informed consent.

To determine the most affected brain regions and for a reproducible definition of reference tissue in MS patients, a database of 6 healthy controls (HC, 3 female and 3 male; mean age 23 ± 6 years; 1 MAB and 5 HAB) was provided by GE Healthcare (The Grove Centre Amersham, UK). The underlying study of healthy subjects was approved by the McMaster University Research Ethics Board. Research was conducted in accordance with the principles of the Declaration of Helsinki and all subjects gave written informed consent.

### Imaging

Dynamic HC PET studies (4 × 30, 3 × 60, 10 × 150, 12 × 300 s) were acquired after injection of 269 ± 7 MBq [^18^F]GE-180 on a Biograph 6 PET/CT (Siemens Healthineers, Erlangen, Germany) and reconstructed with OSEM2D algorithm (8 iterations, 4 subsets, 4 mm Gauss). Standard corrections for CT-based attenuation, scatter, decay, and random counts were applied.

Seventeen dynamic PET studies of 14 RRMS patients were performed on a Biograph 64 PET/CT device (Siemens Healthineers, Erlangen, Germany). Based on previously published experience with HC, a 90-min emission scan was acquired in list mode, starting with injection of 189 ± 11 MBq [^18^F]GE-180. Reconstruction with a 256 × 256 × 109 matrix, voxel size of 1.336 × 1.336 × 2.027 mm^3^ (framing 12 × 10, 4 × 30, 2 × 60, 2 × 120, 16 × 300 s) was performed using the same reconstruction settings as for HC data. PET data were corrected for subject motion within the PMOD Fusion tool (v3.5, PMOD Technologies, Zurich, Switzerland).

For each subject, a T_1_-weighted MRI scan with a slice thickness of at least 3 mm was performed on a Magnetom 3T scanner (Siemens Healthineers, Erlangen, Germany) with intravenous injection of 0.1 mmol/kg contrast agent (Gd-BOPTA, MultiHance; Bracco Imaging, Milan, Italy). Contrast-enhanced (CE) MRI images were co-registered to the corresponding PET data.

### Anatomical brain regions

For VOI-based analysis, anatomical brain regions were defined with the workflow provided within the PMOD Neuro tool (v3.5). First, each PET image was mapped to the corresponding T_1_-weighted CE MRI image by rigid matching using the default settings. Then, each MRI image was normalised to the T_1_-weighted MRI template in Montreal Neurological Institute (MNI) space. This was followed by the application of a maximum probability atlas (Hammers N30R83 [18]) for VOI definition. Grey matter was masked by application of the default threshold of 0.3 on the grey matter probability atlas. Anatomical brain VOIs were then transformed into PET space.

### Reference tissue extraction

SUV was determined at 60 to 90 min p.i. (SUV_60–90_) [11]. For the extraction of brain tissue which is least affected by disease, a voxel-wise comparison of SUV_60–90_ (two sample *t* test) between HC and all MS patient scans was conducted with statistical parametric mapping (SPM8; Wellcome Trust Centre for Neuroimaging, UK) assuming unequal variance. Smoothing of images was not performed. For this purpose, PET data were mapped into MNI space using the corresponding MRI images with the PMOD Neuro tool as described in the previous section. Anatomically defined brain volumes exhibiting a low fraction of significant voxels in SPM were identified by determination of the fraction of voxels with a *t* score above 2.52 (*P* < 0.01) for each volume. Within these volumes, the volume best suited for reduction of variability of healthy tissue uptake was selected by calculating the coefficients of variation of grey matter (GM) and white matter (WM) uptake in HC after normalisation to each eligible brain region.

This was followed by an exclusion of voxels suspected of being affected by disease relying on mean SUV_60–90_ and standard deviation (SD) from HC data in this region. The optimal upper threshold *T*_PRR_ = mean + *a* × SD was iteratively adapted by minimising the difference between the average PRR time-activity curve (TAC) of RRMS patients and the average FC TAC of HC.

### Quantification with DVR and SUVR

Specific binding relative to non-displaceable uptake can be derived directly from compartmental model parameters (binding potential BP_ND_ = k_3_/k_4_). Alternatively, it can be calculated from distribution volume ratios (BP_ND_ = *V*_T_/*V*_ND_ − 1) [19]. Since there is no reference tissue available for [^18^F]GE-180, which is devoid of specific binding, the quantity of interest was specific binding relative to healthy tissue PRR (BP = *V*_T_/*V*_PRR_ − 1 = DVR − 1), which is smaller than BP_ND_ = DVR(1 + BP_ND,PRR_) − 1 [20]. The Logan reference tissue model [21] was used to determine DVR with PMOD Kinetic Modelling tool (v3.4) from dynamic 20–90 min p.i. data [10]. The population average rate k_2_’^REF^ of the reference tissue was set to 0.027 1/min according to the previously published average value for frontal cortex *k*_2_ estimated with one-tissue compartment model [11]. For one exemplary patient, a parametric DVR map was generated from dynamic data reconstructed with a 10-mm Gauss filter. Due to high statistical fluctuations, the coarse filter had to be applied for voxel-wise fitting with Logan reference tissue model.

To assess whether modelling based on dynamic 20–90 min p.i. data can be replaced by values obtained from shorter static scans, a simple quantification based on standardised uptake value ratios (SUVR = SUV_60–90_/SUV_PRR,60–90_) was carried out by comparison with DVR obtained from Logan reference tissue model. Correlation was determined for all brain tissue regions and lesions.

### Segmentation of MS lesions

VOIs of 67 focal MS lesions visible in PET were defined on SUVR images. A delineation method, which aims to find the boundary reproducing threshold *T*_SUVR_ based on the mean signal from 32 hottest voxels of each lesion (SUVR_32Vox_, total volume of 0.116 mL) and affected white matter background (BG) value, was applied [22]:

1 $$ {T}_{\mathrm{SUVR}}=\left({\mathrm{SUVR}}_{32\mathrm{Vox}}-{\mathrm{SUVR}}_{\mathrm{BG}}\right)\times \mathrm{F}+{\mathrm{SUVR}}_{\mathrm{BG}} $$

The fraction *F* = 0.35 was derived from a Nema-NU2-2001 phantom measurement consisting of six hot spheres in BG (1:8) with different volumes (0.5–26.5 mL). The affected WM uptake normalised to PRR averaged over all patients served as BG for the delineation of locally elevated uptake within WM without being influenced by a patient-specific lesion load. Alternatively, background volumes surrounding the focal lesions can be delineated manually for each patient.

### Statistical analysis

Results are presented as mean ± SD. Analysis of group-wise differences between different binding affinity groups and VOI parameters of HC and MS patient data was calculated with Mann-Whitney *U* test (*U* test) using MATLAB (MathWorks, USA), where *P* < 0.05 was considered as a significant difference. Linear correlation of quantitative parameters was performed (Pearson, MATLAB, MathWorks, USA).

## Results

### [^18^F]GE-180 uptake in MS patients

**Fig. 1:**
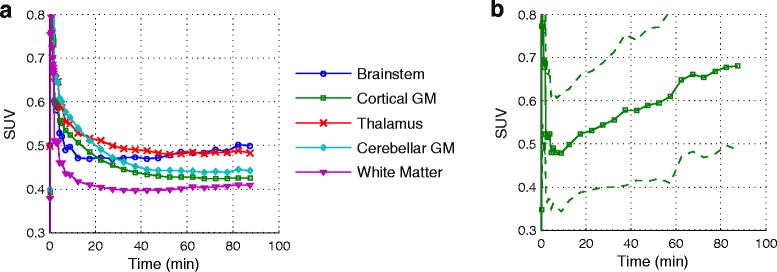
[^18^F]GE-180 time-activity curves in SUV averaged over all MS patient studies. **a** Mean TAC of brainstem (circle), cortical GM (square), thalamus (cross), cerebellar GM (diamond), and white matter (triangle). **b** Mean ± SD TAC averaged over 67 lesions

TAC averaged over all RRMS patient scans are shown in Fig. 1a. [^18^F]GE-180 uptake in brain tissue peaked at about 35 s p.i. with the lowest mean peak-SUV in white matter (0.88 ± 0.3) and the highest mean peak SUV in the thalamus (1.24 ± 0.4) and brainstem (1.16 ± 0.4). Mean peak SUV in cortical and cerebellar grey matter was similar (1.06 ± 0.4, 1.13 ± 0.4). While cortical and cerebellar GM reached a plateau after about 60 min p.i, the brainstem, WM, and also the thalamus of the MS patients exhibited a slowly increasing TAC after the fast wash-out. SUV_60–90_ was lowest in white matter (0.41 ± 0.05), and highest in brainstem (0.49 ± 0.06) and thalamus (0.48 ± 0.05). SUV_60–90_ in cortical GM was 0.43 ± 0.05 and in cerebellar GM 0.44 ± 0.06. In contrast to the uptake kinetics of apparently not affected tissue, MS lesions exhibited a constant increase or saturation of uptake (Fig. 1b) with a mean SUV_60–90_ of 0.7 ± 0.2.

No significant differences (*U* test *P* > 0.05) in SUV_60–90_ were found between MAB and HAB in all anatomical brain regions (e.g. combined frontal, temporal, and parietal cortex SUV: MAB = 0.41 ± 0.04, HAB = 0.41 ± 0.05).

### Reference tissue extraction

**Fig. 2:**
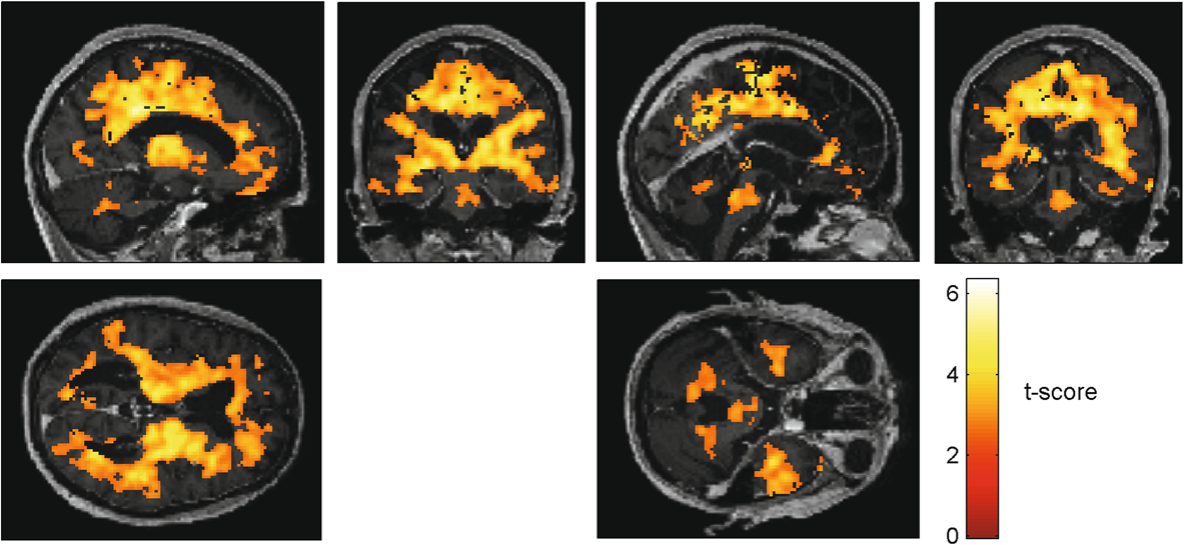
Statistical parametric maps from two sample *t* test with an extent threshold of 60 voxels with *t* score values showing differences in [^18^F]GE-180 uptake in static 60 to 90 min p.i. images between healthy controls and all MS patient scans with a cut-off threshold of *P* < 0.01 in triangular views

Results from SPM group analysis on static 60 to 90 min p.i. images are given in Fig. 2. The *t* score images are given for a cut-off threshold of *P* < 0.01. White matter (average *t* score 2.7 and *P* = 0.03) and the thalamus (average *t* score 2.6 and *P* = 0.06) exhibited the highest fraction of voxels with *P* < 0.01 (> 55%). The fraction with *P* < 0.01 was below 25% in the frontal lobe, temporal lobe, and in cerebellar grey matter (average *t* score < 1.8 and *P* > 0.10). Normalisation to frontal cortex (FC) led to the lowest variability of grey and white matter uptake (GM decreased from 12 to 6%, WM remained at 7%) in HC. Therefore, FC was chosen as the anatomically defined primary reference tissue.

Mean frontal cortex SUV_60–90_ in HC was 0.37 ± 0.04. The optimal upper threshold for unaffected FC voxels obtained by iterative adoption was:

2 $$ {T}_{\mathrm{PRR}}={\mathrm{mean}}_{\mathrm{HC}}+1.7\times {\mathrm{SD}}_{\mathrm{HC}} $$

**Fig. 3:**
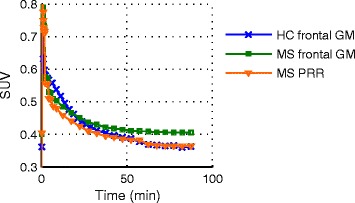
Time-activity curves of frontal cortex in healthy controls (cross), and original frontal cortex (square) and pseudo-reference region (triangle) averaged over all MS patient scans

**Fig. 4:**
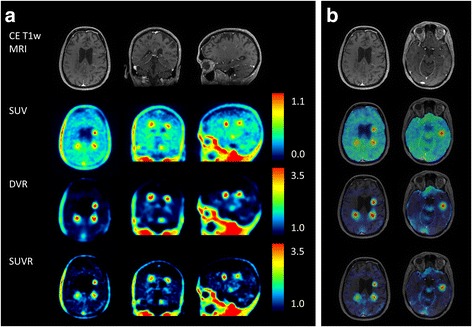
Images of an MS patient in native [^18^F]GE-180 PET space. Top row: T_1_-weighted contrast-enhanced MRI. Second row: SUV image (60–90 min p.i.). Third and bottom row: DVR (from dynamic data reconstructed with 10-mm Gauss filter) and SUVR with lower threshold set to 1.0 for the depiction of specific binding relative to the PRR. **a** Whole head in triangular views. **b** Application of brain mask onto parametric maps for two different transaxial planes with MRI overlay

This corresponds to a SUV_60–90_ threshold of 0.433. The resulting corrected FC volume applied in the following as pseudo-reference region yielded a SUV_60–90_ of 0.37 ± 0.03 averaged over all MS patient studies. No significant difference was found between the corrected frontal cortex SUV_60–90_ in MS patients and the corresponding values in HC (Fig. 3, *U* test *P* = 0.42). Based on this pseudo-reference region, SUVR images were generated as visualised in Fig. 4. Variability in uptake values in patients reduced with PRR normalisation for GM from 11 to 7%, and for WM from 13 to 10%.

### Quantification with DVR and SUVR

**Fig. 5:**
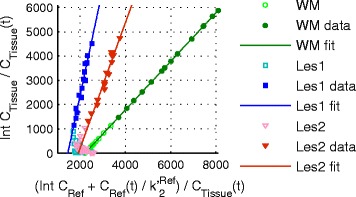
Logan reference tissue plots with t* = 20 min p.i. of white matter (DVR 1.35) and two lesions (lesion 1: DVR 4.5, lesion 2: DVR 2.6) for one exemplary patient

**Fig. 6:**
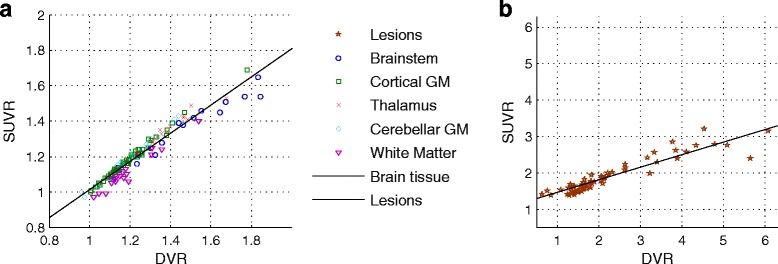
Pearson correlation of SUVR obtained from static 60–90 min p.i. images with DVR from Logan reference tissue model applied to dynamic 20–90 min p.i. PET data. **a** Anatomical brain regions: ρ = 0.97, *P* < 0.001, *y* = 0.79*x* + 0.22. **b** Lesions: ρ = 0.93, *P* < 0.001, *y* = 0.35*x* + 1.12

The linear part of the Logan plot started earlier for brain tissue data than for lesion data and both reached linearity (Fig. 5). DVR derived from Logan reference tissue model showed a very strong correlation with SUVR (Fig. 6: anatomical brain regions ρ = 0.97, *P* < 0.001, and lesions ρ = 0.93, *P* < 0.001). In RRMS patients, thalamus and brainstem exhibited the highest values (SUVR 1.33 ± 0.11 and 1.35 ± 0.17, DVR 1.35 ± 0.14 and 1.45 ± 0.24) and WM and cortical GM the lowest (SUVR 1.11 ± 0.11 and 1.16 ± 0.09, DVR 1.19 ± 0.13 and 1.18 ± 0.10).

For observer-independent assessment of inflammation activity in focal lesions, a SUVR of 1.3 was assumed as affected WM background. This value was derived from SUVR images normalised to PRR by adoption of Eq. (2): *T*_Lesion,BG_ = mean_HC,WM_ + 1.7 × SD_HC_, where *T*_Lesion,BG_ served as lower threshold for the definition of affected white matter voxels in MS patients. For all RRMS patients studied, the average SUVR of lesions delineated by this method was between 1.3 and 3.2 (mean 1.9 ± 0.5). Maximum SUVR within lesions ranged between 1.5 and 4.9 (2.4 ± 0.9). All MS lesions exhibited an increasing or saturating TAC (Fig. 1b).

## Discussion

This study aimed to provide a robust, clinically suitable quantification approach for the third-generation TSPO ligand [^18^F]GE-180 in MS patients. The investigated static 60–90 min imaging containing a PRR-based SUVR quantification correlated well with DVR from modelling by application of the Logan reference tissue model on dynamic 90 min and thus proved suitability for clinical TSPO PET application, when patient compliance and economic aspects have to be considered. The presence of non-saturated lesion TACs suggests that a prolongation of the scan duration, at the cost of a lower count statistic, might allow for an improved assessment of equilibrium and tracer wash-out.

Binding potentials derived with reference modelling (BP = DVR − 1) reported previously for the prototypic TSPO ligand [^11^C](R)-PK11195 in healthy controls, and MS patients were in a similar range as results presented here for [^18^F]GE-180: the lowest BP was found in normal-appearing white matter and the highest BP in the thalamus and the brainstem [23–25]. In agreement with our SPM analysis results, PET signal was significantly elevated compared to HC in contrast-enhancing lesions, thalamus, parts of the brainstem, and in white matter frequently following white matter fibre tracts [23, 25–27].

MS lesions exhibited a high [^18^F]GE-180 uptake and contrast, enabling a visual detection of focally elevated tracer accumulations (Fig. 4). The lesion-to-WM-background ratio (up to a threefold increase in mean lesion SUVR) appears to be high for [^18^F]GE-180 compared to other TSPO radioligands previously used in MS patients [23–30]. [^11^C]PK11195 signal in static images normalised to cortical grey matter was significantly higher in lesions with CE in MRI compared to normal white matter (up to a factor of 1.4) [26]. For [^18^F]FEDAA1106, lesions with CE in MRI were not detectable in SUV and Logan *V*_T_ images, probably due to a high non-specific uptake [30]. Both [^18^F]PBR111 and [^11^C]PBR28 showed an increased *V*_T_ in some lesions with CE in MRI [24, 28, 29]. However, for [^11^C]PBR28, static SUV_90–120_ images were too noisy for visual detection of MS lesions, most probably due to the short half-life of ^11^C in combination with a high-resolution PET tomograph [29].

The critical aspect for robust and reliable lesion quantification is the choice of the reference region. It is difficult to propose a standard reference region for all neurological diseases since patterns of affection vary widely. In MS, immune cell infiltration is predominantly localised in focal white matter lesions. However, as the disease progresses, inflammatory changes spread throughout the CNS and no region can be assumed to be unaffected. The corrected frontal cortex seems to be a suitable pseudo-reference region, at least for RRMS patients, since grey matter was reported to be less affected than white matter in early stages of MS [24–26, 28]. In order to identify the least affected regions in RRMS patients, we compared our patients with a group of young HC, in which no CNS inflammation should be present. Although the SPM analysis revealed a non-negligible fraction (24%) affected by disease in the frontal cortex, time activity curves were not significantly different for HC and RRMS patients in this region, and it was feasible to exclude suspicious voxels in this relatively large and well-defined region. The VOI-based comparison of PRR SUVs with HC data showed good concordance. Normalisation of PET data by the corrected frontal cortex uptake reduced inter-patient variability in grey (from 11 to 7%) and white matter (from 13 to 10%) signals.

Alternative methods recommended for reference tissue TAC generation of TSPO tracers are data-driven clustering (DC) [31, 32] or supervised clustering (SC) on dynamic data preselected with a brain mask [33, 34]. SC has been validated for [^11^C](R)-PK11195 and also tested for [^18^F]GE-180 [8]. However, DC and SC require dynamic PET studies and previous publications show that SC might not sufficiently exclude affected voxels in some cases and that other methods might be superior for the exclusion of affected voxels [8, 35]. A promising approach using fixed thresholds for the definition of affected voxels was applied to BP images derived with SC reference tissue [23] but also needs dynamic imaging.

The reported high inter- and intra-subject variability found for second-generation radioligands in other studies was attributed to differences in binding affinity status and in plasma protein binding [5, 36, 37]. For [^11^C](R)-PK11195, in vitro and in vivo data show no significant differences between binding affinity groups. Unpublished in vitro work by D. Owen with cold GE-180 displacing [^3^H]PK11195 has shown a binding affinity ratio of 15:1 between HABs and MABs [11]. However, Feeney et al. [11] found no significant differences between MABs and HABs similar to [^11^C](R)-PK11195 in healthy brain tissue of human subjects [16]. This is in line with the results of our current study in which we found no differences between MAB and HAB. Although in vitro prediction of differences in specific binding can differ from the relation observed for in vivo data [15, 38], it is questionable whether this can explain the results. Such a discrepancy may be explained by the high dependency of the in vitro studies on experimental conditions like temperature, fluid composition, and presence of intact mitochondria [15]. Furthermore, brain microvascular endothelial cells change BBB properties in vitro [39]. Fan et al. [10] suggest that the finding that no differences could be observed in vivo may be caused by a lower TSPO affinity of [^18^F]GE-180 compared to other second-generation TSPO tracers. Another explanation proposed by both previous studies is the low brain tissue uptake [10, 11]. The high fraction of ligand bound to plasma proteins, probably resulting from a relatively high lipophilicity (logD at pH 7.4 is 2.95 [40]), may be the reason for the slow propagation into tissue and the constantly high activity concentration in blood vessels dominating signal in healthy tissue (suggesting similarities to [^11^C](R)-PK11195 in vivo) [41, 42]. Another reason for low uptake in brain may be a fast clearance by efflux pumps.

Yet, we observe a high contrast in MS lesions and also in gliomas as published recently [43]. The important question is what are the underlying processes leading to this contrast. Does it reflect specific binding to TSPO or rather other processes like a BBB breakdown? For gliomas, we could demonstrate that [^18^F]GE-180 uptake patterns do not correlate with contrast enhancement in T_1_-weighted MRI images and that even for some gliomas, the highest [^18^F]GE-180 uptake can be found in non-contrast-enhancing tumour areas [43]. Still one might hypothesise that those areas exhibit micro BBB breakdown without apparent enhancement of contrast agent in MRI [44], which may allow the passage of [^18^F]GE-180 through the leaky BBB. However, we also observe areas with CE in T_1_-weighted MRI, i.e. with BBB breakdown, but without elevated [^18^F]GE-180 uptake [43]. Even if micro BBB breakdown might ease the supply of [^18^F]GE-180, the [^18^F]GE-180 signal intensity does not correlate with severity of BBB breakdown, leading to the assumption that the dominant process resulting in the observed high range of [^18^F]GE-180 binding should be attributed to TSPO expression levels rather than mere BBB breakdown. Nevertheless, the lack of micro BBB breakdown in some regions may lead to an underestimation of TSPO expression.

One limitation of this study may be the usage of data from two different PET/CT devices in which the PET part is identical, but the CT data may yield a slightly different attenuation correction. Also, for future clinical studies, it would be beneficial to gather a larger database of HC with varying age to account for age-related changes. Furthermore, for an encompassing and comprehensive interpretation of the underlying processes, it is indispensable to perform in vivo blocking studies in combination with pharmacokinetic modelling with a metabolite corrected arterial input function and a longer scan duration.

The possibility of static PET imaging provided by the proposed method in contrast to dynamic PET imaging will greatly increase the acceptance by patients, as 30-min scans are usually well-tolerated and the imaging protocol does not include blood sampling, which is often perceived as an invasive, displeasing method by patients and is therefore often avoided in clinical settings. With these tools, TSPO PET with [^18^F]GE-180 may enable straightforward clinical assessment of neuroinflammatory activity in MS beyond the scope of structural MRI and seems to be a highly promising imaging method to assess disease activity and therapy response in RRMS patients.

## Conclusions

In patients suffering from RRMS, the new TSPO ligand [^18^F]GE-180 presented a highly elevated signal up to a threefold increase in SUVR of focal lesions compared to surrounding background. Our data demonstrate a high correlation between parameters obtained from dynamic PET imaging with simple SUV ratios extracted from static 60–90 min [^18^F]GE-180 PET scans using the corrected frontal cortex as pseudo-reference region.

## Abbreviations

BBB: Blood-brain barrier
BP: Binding potential
CE: Contrast-enhanced
DC: Data-driven clustering
DVR: Distribution volume ratio
FC: Frontal cortex
GM: Grey matter
HAB: High-affinity binder
HC: Healthy controls
MAB: Medium-affinity binder
MNI: Montreal Neurological Institute
MRI: Magnetic resonance imaging
MS: Multiple sclerosis
PET: Positron emission tomography
PRR: Pseudo-reference region
RRMS: Relapsing remitting multiple sclerosis
SC: Supervised clustering
SD: Standard deviation
SUV: Standardised uptake value
SUVR: Standardised uptake value ratio
TAC: Time-activity curve
TSPO: Translocator protein
*V*_T_: Volume of distribution
WM: White matter
